# Comparative Effectiveness of Two Nonsurgical Treatments to Reduce Oral Health Disparities From Untreated Tooth Decay in Older Adults: Protocol for a Cluster Randomized Trial

**DOI:** 10.2196/17840

**Published:** 2020-09-08

**Authors:** Suchitra Nelson, Jeffrey M Albert, Peter Milgrom

**Affiliations:** 1 Case Western Reserve University Cleveland, OH United States; 2 Department of Psychiatry and Behavioral Sciences University of Washington Seattle, WA United States

**Keywords:** dental caries, older adults, atraumatic restorative treatment, silver diamine fluoride, fluoride varnish

## Abstract

**Background:**

The majority of dental caries lesions in older adults are at the gumline, at the edges of failed fillings and crowns, and in the surfaces of roots after gum recession. These lesions are difficult to restore with conventional surgical treatments using a dental drill and restorations often fail. Clinical guidelines are general and apply treatments that were designed for younger individuals in the dental care of older adults.

**Objective:**

This study will compare the effectiveness of 2 evidence-based nonsurgical strategies to manage dental caries lesions in adults aged 62 or older: (1) biannual topical application of silver diamine fluoride versus (2) atraumatic restorative treatment + biannual fluoride varnish.

**Methods:**

A cluster randomized clinical trial is being conducted in 22 publicly subsidized and other low-income housing facilities/sites (Arm 1: 11 sites, 275 participants; Arm 2: 11 sites, 275 participants). At baseline, participants will be screened for caries lesions. Those with nonurgent lesions will be treated according to the treatment arm to which the housing site was randomly assigned. The primary outcomes are caries lesion arrest, tooth sensitivity, and tooth pain at 52 weeks after treatment. Analytic methods for the primary aim include a generalized estimating equation approach to determine noninferiority of silver diamine fluoride relative to atraumatic restorative treatment + fluoride varnish treatment.

**Results:**

The trial was funded in April 2019. Enrollment began in September 2019 and results are expected in June 2023.

**Conclusions:**

This study will inform the standard of care for treating caries lesions in older adults. If effective, either of these interventions has broad applicability in clinical and community-based settings.

**Trial Registration:**

ClinicalTrials.gov NCT03916926; https://clinicaltrials.gov/ct2/show/NCT03916926

**International Registered Report Identifier (IRRID):**

DERR1-10.2196/17840

## Introduction

Over 96% of US adults aged 65 or older have had at least one caries lesion (cavities or tooth decay) in the permanent teeth [1]. About 37% of adults aged 65 or older have tooth decay in exposed root surfaces [1]. Dental caries is the primary cause of tooth loss in older adults. Further, untreated tooth decay is disproportionately found in minority and low-income older adults [2]. Consequently, tooth loss is also significantly higher in non-Hispanic Blacks compared with other groups [2], who may also have vulnerable root surfaces because of untreated periodontal diseases [3].

The progression of oral diseases adversely affects general health [3] and individuals with tooth loss reported poorer oral health-related quality of life [4]. A 2018 oral health screening and survey of older adults highlighted that many, especially those with low incomes, are living with significant untreated dental disease with impacts on chewing, nutrition, and overall well-being that requires immediate attention and focus [5].

A recent study of 202 older adults from 16 publicly subsidized housing units in Northeast Ohio found that the proportion with untreated caries lesions (58%) is twice the national average (28%) [6]. These 202 older adults reported the following symptoms: 40% with tooth pain, 54% with tooth sensitivity, 23% with bleeding gums, 35% with loose teeth, and 67% with dry mouth [7]. All had 1 medical condition and 83% had 2 or more medical conditions [7]. Nationally, 92% of US older adults are reported to have at least one medical condition, and 77% have 2 or more conditions [8]. Poor oral health contributes to worsening general health in older adults [9-15].

Conventional restoration of cavities in older adults is more complex than in younger individuals because of age-related changes to the enamel, dentin, and pulp chamber [16]. The majority of such cavities occurs at the gumline, at the periphery of failed fillings and crowns, and in root surfaces after gum recession [17]. These cavities are difficult to restore with conventional surgical treatments using a dental drill and the restored lesions often fail [18]. A randomized trial in older adults with root caries lesions found similar restoration survival rate (87% vs 91%) with atraumatic restorative treatment (ART; using high-viscosity glass ionomer cements [GICs]) versus conventional fillings, respectively [19]. Professional applications of sodium fluoride varnish (FV), thymol chlorhexidine varnish, and silver diamine fluoride (SDF) are also effective in preventing new lesions or arresting existing ones [20]. Annual professional applications of topical 38% SDF were effective in arresting and preventing root caries lesions among older adults in clinical trials [21-23]. A systematic review and meta-analysis support the use of SDF [24,25], the use of ART with high-viscosity GICs [26], and the use of 5% FV [27] for caries management in older adults. However, there have been no trials of comparative effectiveness of nonsurgical caries intervention strategies

Therefore, the objective of this randomized clinical trial is to compare 2 nonsurgical evidence-based strategies for untreated tooth decay: SDF versus ART + FV to improve clinical (caries lesion arrest) and patient-reported (tooth pain, sensitivity) outcomes among community-dwelling older adults. Both intervention arms can be delivered by dental hygienists in alternative (nondental clinic) settings. *Nonsurgical* in this context means treatments that do not require the use of a dental drill. The reason this is a disparity issue is that the field of dentistry lacks specific standards of care for older adults, especially those who have low income, with multiple medical problems, physical frailty, and dementia. Instead, the typical dentist tries to apply surgical treatments developed for younger, healthier individuals, with predictably poor results.

## Methods

### Trial Design

This is a multisite, single-blind, parallel-arm, community-based cluster randomized controlled trial with 2 arms. The trial has been registered with Clinicaltrials.gov (NCT03916926) and is currently in the recruitment phase. This protocol report follows the SPIRIT [28] and CONSORT (Consolidated Standards of Reporting Trials) guidance [29].

### Research Objectives and Hypothesis

The primary aim is to compare 2 evidence-based strategies in low-income older adults aged 62 or older followed for 52 weeks (1 year): a “simple medical” strategy of topical application of SDF (Arm 1) versus a “typical dental” strategy consisting of ART and topical application of FV (Arm 2). The rationale for adding FV to the ART arm is that ART has a direct effect on the treated tooth and perhaps on the adjoining tooth surface while FV has whole mouth benefits, as does SDF. Our primary hypothesis is that simple medical treatment (Arm 1) is not inferior to typical dental treatment (Arm 2) for clinical (caries lesion arrest) and patient-reported (tooth pain/hypersensitivity) outcomes at 52 weeks after treatment. Our secondary hypothesis is that the simple medical treatment (Arm 1) is not inferior to typical dental treatment (Arm 2) for clinical (new caries lesions) and patient-reported (oral health quality of life) outcomes at 52 weeks after treatment.

### Participant Recruitment, Enrollment, and Retention

Study sites are 22 housing facilities for adults of low income in Northeast Ohio. In these facilities, residents live independently in individual apartments. Older adult tenants aged 62 or older will be approached for participation. Those who self-report that they are without teeth will not be recruited. The recruitment will follow a 2-stage process: (1) all participants consented will participate in the dental screening; (2) participants found to have nonurgent cavities (ie, those without irreversible pulpitis, periapical abscess or cellulitis or facial swelling) at the screening and who fulfill the inclusion criteria (see below) will be enrolled into the clinical trial.

Service coordinators at the facilities will serve as site liaisons. The study principal investigator/project manager will provide the coordinators with an introductory letter/flyer (Multimedia Appendix 1) containing study information and ask that it be given to tenants and posted in public areas. The coordinators will arrange an informational meeting (study dentist will give a talk on the interventions as suggested by our stakeholders), and study staff will present information regarding participation at scheduled group events such as tenant meetings or health fairs. For planning purposes, coordinators will have a sign-up sheet for those interested in the sessions. Study staff will schedule potential participants for one-on-one sessions at the housing facility to obtain informed consent and collect a baseline survey. A dental examination and treatment appointments will then be scheduled at each facility according to a designated roll-out schedule for each facility, approximately 1-2 weeks following consent and baseline data collection.

According to the 2-stage process for enrollment, the *initial* *inclusion criteria* of participants are as follows: provide signed and dated consent form, willing to comply with all study procedures, being available for the duration of the study (1 year), male or female, aged 62 or older, and living in a participating facility. In the second stage of enrollment, *additional inclusion criteria* for continuation and participation in the randomized controlled trial are based on the dental screening. A participant must have at least one untreated root surface or coronal caries lesion on any permanent tooth with an International Caries Detection and Assessment System (ICDAS) II [30] active lesion score of 3 or greater (localized enamel cavity to extensive cavity). At this stage, further *exclusion criteria* are sensitivity to silver or other heavy-metal ions or oral ulcerative gingivitis or stomatitis, which prevents the potential participant from receiving study treatment. The study has a 26-month recruitment time frame that started in October 2019.

**Figure 1:**
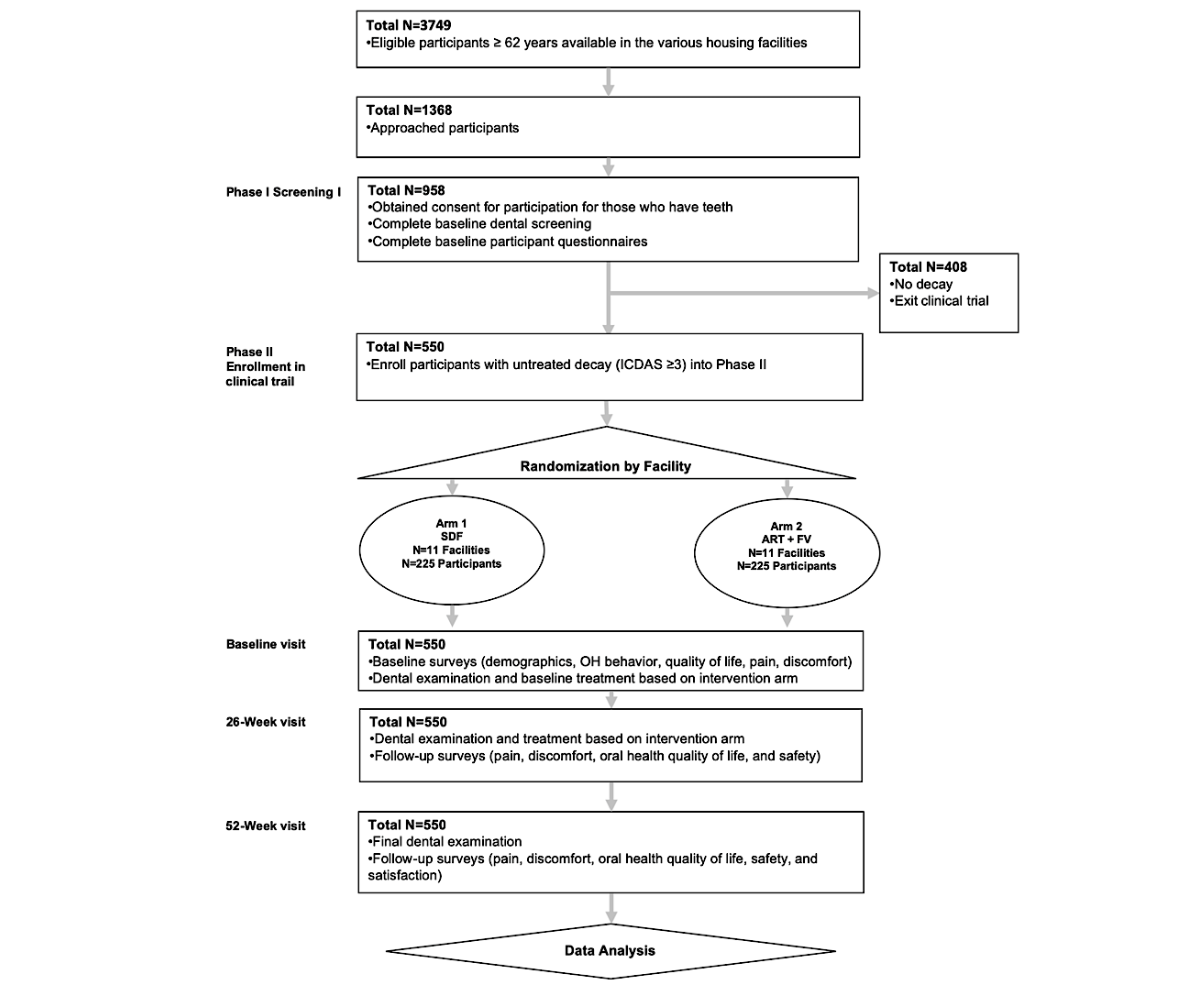
Study design.

Participants are followed at 26 weeks (6 months) and 52 weeks (12 months) that includes dental examinations/treatment and survey completion. This follow-up period is consistent with another caries arrest trial [21]. The schematic of the study design is presented in Figure 1.

All visits will occur at the housing facility where the individual participant resides. Several strategies will be used to retain the participants: promotional items (ie, pens, magnets) with the study logo and contact phone number at recruitment (alternate contact information for family/friends that may allow to reach the participant if primary contact information becomes invalid will be obtained at recruitment); annual birthday/holiday cards will be sent to participants to maintain contact; and newsletters will be sent twice a year with updates on the study’s progress including reminders to update their contact information (by phone or postal mail). Assistance from service coordinators or other facility staff will be sought to maintain contact with hard-to-reach/contact participants. Cash incentives will be given to the participants at the baseline screening and examination (US $25); those enrolled in the clinical trial will receive additional incentives at 26-week follow-up visit (US $40), and at the 52-week visit (US $40) for the completion of all study procedures.

The Case Western Reserve University Institutional Review Board approved all study procedures (STUDY20190481) and written informed consent is obtained from the participant.

### Interventions

#### Conceptual Model and Design

**Figure 2:**
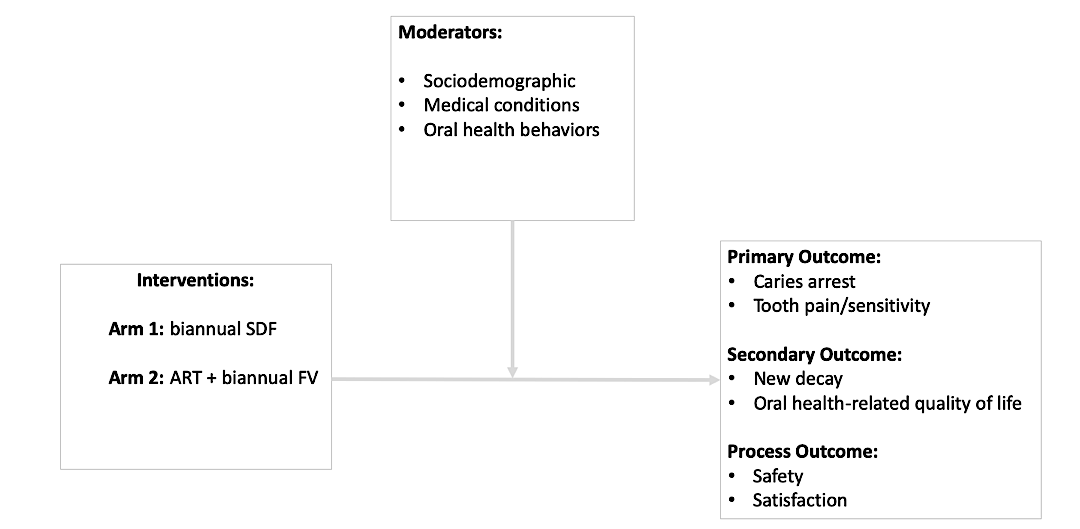
Conceptual Model.

Figure 2 shows the study model for the intervention. The comparative effectiveness of “simple medical” (ie, SDF) versus “typical dental” (ie, ART + FV) intervention in this study is focused on the person level to address the unique oral health needs of low-income older adults. Figure 2 indicates that the proposed intervention is hypothesized to arrest caries in older adults with untreated caries and prevent tooth pain/hypersensitivity (primary outcomes), and prevent new decay and improve oral health-related quality of life (secondary outcomes) over a 52-week follow-up period. Safety and satisfaction measures are process outcomes that are critical to assess for sustainability and dissemination of the interventions. Factors likely to moderate the effectiveness of interventions are sociodemographics, chronic medical conditions, and oral health behavior. These baseline (pretreatment) variables are also considered as prognostic variables (related to outcomes).

#### Specifics of the Intervention

##### Arm 1

The treatment will be biannual application (at baseline and 26 weeks) of topical 38% SDF (Advantage Arrest; Elevate Oral Care, LLC) following manufacturer’s instructions and published guidelines [31].

##### Arm 2

The treatment will be ART [19] with the cavity restored at baseline with resin-reinforced GIC (GC Fuji Automix LC; GC America Inc). Participants in this arm will also receive biannual (at baseline and 26 weeks) topical FV application (FluoriMax 2.5% NaF Varnish; Elevate Oral Care, LLC) applied according to manufacturer’s instructions.

#### Administration of Intervention

The treatments are administered by licensed dental hygienists supervised by a study dentist. All nonurgent cavitated lesions will be considered for treatment in both arms. For application of ART, if there are too many teeth to be treated, then priority will be in this order: (1) anterior teeth; (2) any tooth retaining an appliance (eg, partial denture); (3) teeth that are in occlusion such as biting teeth. The procedures are presented in the following sections

##### Arm 1

SDF will be applied to all carious lesions at the baseline visit and the 26-week visit. The cavity will be cleaned with a tooth brush to remove debris, isolated with cotton rolls, and then SDF from a single-use ampule will be applied using the manufacturer-supplied brush. The lesion will be allowed to air dry for about 1 minute. The participants can then resume normal activity with no restriction.

##### Arm 2

ART will be administered only at the baseline visit, but FV will be applied biannually at the baseline and the 26-week visit. First*,* the cavity will be isolated with cotton rolls. The dental hygienist will remove debris with moistened cotton pellets. Then, soft demineralized tooth structure will be removed with a spoon excavator at the periphery of the lesion by the supervising dentist, where the shape of the cavity permits access with the spoon excavator. A manual tooth brush and plain pumice will be used to remove any further loose material. The cavity will be washed with water. Second*,* the lesion will be conditioned with the manufacturer-supplied polyacrylic acid for 10-15 seconds, rinsed with water, and then resin-reinforced glass ionomer restorative will be inserted into the cavity using the manufacturer’s delivery device and coated with a protective gel. The restoration will then be polymerized for 20 seconds using a manufacturer-supplied visible light. Finally, FV will be applied to all tooth surfaces using the manufacturer’s brush system. The material will be allowed to dry for 3-5 seconds. The patient will be instructed to avoid eating or dental hygiene for 4 hours.

#### Procedures for Training Interventionists and Monitoring Intervention Fidelity

The study interventionists (dental hygienists) will receive didactic and clinical instruction in the application of SDF, ART, and FV from 2 gold-standard experts who have been recognized as national academic and clinical leaders in the application of these interventions. The hygienists will undergo a 2-day training and calibration exercise with extracted teeth and applying the intervention on patients. To be certified, the study hygienist must demonstrate mastery: by completing at least three ART procedures on older adult patients with cavities and at least one SDF and FV treatment on an older adult patient; and also complete a written examination on procedures.

To ensure study treatment is delivered per protocol and meets the requirements of Ohio law, a study dentist will be present for supervision. If there is a problem with the delivery of the treatment or adherence to protocol, then the hygienists will be given corrective training. In addition to direct supervision, a research staff will take random images of ART restorations. A digital camera (Canon EOS Rebel T2i 550D, Canon Inc.) fitted with a 100-mm macro-lens and a ring flash will be used. The research staff have been trained in using the camera. The research staff randomly selects from among the participants receiving ART treatment on any particular day. These clinical photographs will be reviewed on a quarterly basis by the supervising dentist and corrective training will be provided to the hygienists if there is a problem.

Another third gold-standard expert (with expertise nationally and internationally) will calibrate/train the examiners in the ICDAS protocol in a separate 4-day training session. This will include a didactic presentation and clinical examination of a few older adult patients with the instructor. The calibration session consists of the gold standard expert and the hygienist examining 10-15 older adult patients separately to calculate interrater reliability. The hygienist and dentists completed these training sessions in August 2019 prior to participant recruitment.

Dental examinations will be conducted in a portable dental chair in the housing facility. A total of 4 examiners have been trained. The interexaminer reliability was good to excellent for ICDAS lesion severity (wκ=0.62-0.68), lesion activity (wκ=0.62-1.0), and fillings (wκ=0.78-0.86), respectively. Examiners will not utilize dental radiographs. Examiners will be recalibrated before follow-up. At follow-up, the examiner will also not have access to the results of the first examination to avoid detection bias. A study dentist is present at each housing facility to supervise the examinations and treatment.

### Data Collection

Participants’ schedule for data collection is given in Table 1. A summary of the data collected is as follows.

**Table 1:** Summary of study measures and timeline in the older adults trial.

Variable type, measure, and scale			Source	Timeline
**Intervention by study arms**				T_0_^a^, T_26_^b^
	Arm 1: Biannual SDF^c^			
	Arm 2: ART^d^ + biannual FV^e^			
**Primary outcome**				
	**Clinical dental examination**			T_0_, T_26_, T_52_^f^
		Caries arrest, frequency of treated surface/teeth that are arrested (%)	ICDAS^g^ coronal and root [30]	
	**Self-reported evaluation**			T_0_, T_26_, T_52_
		Tooth pain/sensitivity, overall score	PROMIS version 1.0 – Pain Intensity 3a (Modified for dental) [32] and Dental Discomfort Questionnaire (Modified for adults from [33])	
**Secondary outcomes**				
	**Clinical dental examination**			T_0_, T_26_, T_52_
		New decay, frequency of new decayed surface/teeth (%)	ICDAS coronal and root [30]	
	**Self-reported evaluation**			T_0_, T_26_, T_52_
		Oral health quality of life, GOHRQoL^h^ overall score	GOHRQoL [34]	
**Process outcome**				
	Safety, frequency of adverse events (%)		Safety Questionnaire (Adapted for Adults from [35])	T_0_, T_26_, T_52_
	Satisfaction, overall score		TSQM^i^ (Modified for dental) [36] and Satisfaction with new treatment for cavities (Modified for adults from [37])	T_52_ (for both satisfaction measures)
**Moderators**				
	Sociodemographic, frequency (%)		NHANES^j^ III [38]	T_0_
	Medical condition, frequency (%)		Common chronic health condition for adults 65+ (Adapted from [39])	T_0_
	Oral health behavior, overall score		Oral hygiene (Adapted from [40,41])	T_0_
	Oral health symptoms, overall score		Self-reported measures of current oral disease/tissue damage (Adapted from [42])	T_0_

^a^T_0_ = 0 week/Baseline visit/Baseline visual/tactile dental examination (Arm 1: SDF, Arm 2: ART + FV)

^b^T_26_=26-week follow-up visit/Visual/tactile dental examination (Arm 1, Arm 2).

^c^SDF: silver diamine fluoride.

^d^ART: atraumatic restorative treatment.

^e^FV: fluoride varnish.

^f^T_52_=52-week final visit/Visual/tactile dental examination (final).

^g^ICDAS: International Caries Detection and Assessment System.

^h^GOHRQoL: Geriatric Oral Health Quality of Life.

^i^TSQM: Treatment Satisfaction Questionnaire for Medication.

^j^NHANES: National Health and Nutrition Examination Survey.

#### Outcome

The primary outcomes are (1) clinical outcomes (caries lesion arrest [ICDAS activity code 1], inactive); and (2) participant-reported outcomes (tooth pain and hypersensitivity). Clinical outcomes will be assessed through dental examinations conducted by calibrated examiners. Participant questionnaire will assess patient-reported outcomes using validated instruments (Table 1). All primary outcomes will be measured at baseline and at 26 and 52 weeks.

The secondary outcomes are clinical (new caries lesions, ie, ICDAS lesion codes ≥3 on any surface that was previously sound), as assessed by dental examination; and participant-reported outcomes (oral health quality of life) assessed through questionnaires. All secondary outcomes will be measured at baseline and at 26 and 52 weeks.

#### Other Moderator and Process Measures

The moderator variables include questions regarding sociodemographics [43], medical/physical conditions [44], oral health behavior [40,41], and oral health symptoms [42] that will be collected at baseline (prior to treatment) through the participant questionnaire. The process outcomes include questions regarding safety [35] assessed via a measure that will be collected at pretreatment and after treatment following the baseline treatment, and at 26 and 52 weeks; and satisfaction survey [36,37] with the treatment assessed after the exit visit at 52 weeks.

Study data will be collected and stored using the REDCap Electronic Data Capture platform hosted by Case Western Reserve University. The study staff use tablet computers for on-site data entry.

### Fidelity Checks

The study staff members who will facilitate recruitment, scheduling, and data collection will attend a 2-day in-person training on the conduct of the study protocol and logistics. Specialized training in interviewing older adults will also be included. In-class training incorporates the topics of human subject protection, good clinical practice, and the study protocol. Study staff members will be monitored through periodic data audits and through direct observation of calls and recruitment activities. Staff members will receive feedback on their performance and conduct including but not limited to these specific areas, adherence to protocol and good clinical practice. Corrective training will be provided as required.

### Sample Size and Power Estimates

This study tests the hypothesis that simple medical treatment (Arm 1) is noninferior to typical dental treatment (Arm 2) for primary outcomes (caries arrest, tooth pain/hypersensitivity) and secondary outcomes (new decay, oral health quality of life) at 52 weeks after treatment. For continuous outcomes power was computed using a variance correction (ie, variance inflation factor) to take into account possible correlations of outcomes within cluster. All computations of required effective samples sizes (or corresponding power) were done using the PASS 2005 software. For binary outcomes, a specialized program for cluster randomization (a noninferiority test comparing two proportions) was used.

The first primary outcome tooth pain was defined as change in pain (based on a 100-mm visual analog pain scale, where a higher score indicates worse pain) from baseline to 1 year. A mean difference of 0 between the SDF and ART + FV arms, and noninferiority to be within a margin (mean difference) of 8 between the SDF and ART + FV arms were considered, and based on prior literature we also assumed a common standard deviation of 25 [45]. Further, an average of 25 patients recruited per site, an intraclass correlation of 0.01 (based on prior studies and literature for similar populations) [46,47], and a conservative 15% dropout rate (based on prior studies) were considered including the possibility of deaths among participants. The use of a .025 α-level one-sided *t* test to test the null hypothesis of inferiority versus the alternative hypothesis of noninferiority (as defined above) in 11 sites per treatment group (corresponding to 275 subjects per treatment group or 550 in total) will provide an estimated 89% power to conclude noninferiority.

A second primary outcome is arrest rate. Previous data [19,48] show a high arrest rate (of around 90%) for ART + FV. A similar arrest rate for biannual application of SDF is expected [23]. For the power calculation, the unit of analysis was assumed to be the person, and considered the binary outcome of *arrest*, which is defined as all lesions for an individual being arrested. An equal (person-level) arrest rate of 90% for the ART + FV and SDF groups, and noninferiority to be within a margin (difference in arrest proportions for ART + FV versus SDF) of 0.09 were considered. With the same assumptions as before using a .025 α-level one-sided *z* test, the targeted sample size of 275 subjects in 11 sites per treatment group will provide an estimated 85% power to conclude noninferiority.

### Randomization and Blinding

Randomization is at the level of the cluster (housing facility) for logistical efficiency, that is, we require supplies and personnel only for one intervention (rather than both) at each site. In addition, having only one intervention at each site will greatly reduce the potential for error (mistakenly giving the wrong treatment) that could otherwise occur with people at the same site assigned to different treatments. Furthermore, keeping the same treatment at each site reduces chances of *contamination* (ie, participant discussing their treatment with others). Particularly as one of the primary endpoints (self-reported pain/sensitivity) is subjective, it is important, in a context where blinding is not possible, to minimize the possibility that participants may perceive, through communication with others, that the treatment they are receiving is inferior or superior.

Additionally, stratified cluster randomization will be used, that is, a block (constrained) randomization approach in which balance over treatments is assured for 2 key cluster-level (stratification) variables, namely, facility size (>100 versus ≤100 residents) and geographic location (Cuyahoga County vs other). While the randomization is at the housing level, the study objectives, interventions, and primary outcomes all pertain to the individual level.

Study participants are blinded to the study group. Study staff will be blinded at the time of recruitment and obtaining consent.

### Planned Analysis: Primary and Secondary Outcomes

Each primary outcome will be compared between the SDF and ART + FV groups. For tooth pain, a 95% CI based on a *t* test for the difference in mean responses (SDF minus ART + FV) will be computed. If the CI lies within the interval (–∞, 8), we may conclude *noninferiority* of SDF relative to ART + FV treatment. The CI may secondarily be examined to assess possible superiority of one intervention over the other. For arrest rate, a 95% CI for the difference in rates (based on a *z* statistic) will be computed. If the CI lies within the interval (–0.09, 1), we may conclude *noninferiority* of the SDF relative to ART + FV treatment. As above, possible superiority of one intervention over the other may also be assessed. For other outcomes, we will also compute 95% CI for differences in means (or proportions for binary outcomes). These secondary outcomes will be assessed in an exploratory manner for possible superiority or inferiority based on appropriate margins.

We will seek to corroborate initial results using a generalized estimating equation (GEE) approach. For each outcome, a GEE (marginal) model will be fitted that includes a treatment indicator and prognostic variables (including sociodemographic variables, medical conditions, and oral health behaviors). Appropriate link functions (eg, logit link for binary outcomes and identity link for continuous outcomes) will be specified and an exchangeable working correlation matrix will be used to allow for correlations within site. The arrest outcome will be analyzed as a binary outcome (as described in the “Sample Size and Power Estimates” section), and secondarily as the number of arrested lesions assuming an appropriate distribution (eg, negative binomial) and link function (eg, log link). Robust *t* tests with correction for a small number of clusters [49] will be used to test for treatment effects and corresponding 95% CIs computed.

Secondarily, the above GEE approach will be extended to analyze the repeated (baseline and 26 and 52 week) measures for each outcome. The models for each outcome will include the same prognostic variables as before, as well as time and a time × treatment interaction, and will allow for correlations among the repeated measures (eg, using a first-order autocorrelation structure). If a substantial within-facility correlation is found, the need to incorporate facility as a second cluster level (within which a person [ie, the first cluster level] is nested) will be assessed. To compare trends over time for the 2 interventions, estimation and testing (via a robust *t* test) for the interaction term will be done. If the use of 2 cluster levels is not found to be feasible in the GEE approach, a generalized mixed effects model approach will be considered.

### Dissemination

The study results will be disseminated through local and national conferences, scientific publications, and channels established by a 11-member stakeholder engagement group established for this project. For local and city levels, dissemination will occur through presentations, workshops, and educational programs conducted in community organizations. At the state level, results will be shared with the Ohio Department of Health, Medicaid policymakers, and third-party payers who can revise reimbursement policies. Nationally, Housing and Urban Development (HUD) will create flyers to be distributed to their housing facilities and share results with other states. All stakeholder partners have suggested sharing the results on their organization’s respective webpages and newsletters, which reach large national, state, and local audiences.

### Data Sharing Statement

The nonidentified data files, data dictionary, and supporting documentation will be available without restriction by application to the project biostatistician.

## Results

Recruitment started in October 2019 and is currently ongoing (expected to be until the end of 2021). All follow-up intervention and data collection will be completed by the end of 2022. Final results are expected in June 2023.

## Discussion

### Overview

Dental practitioners lack robust guidance and standards of care for the treatment of older adults. The ability to provide treatment and the treatments themselves are impacted by the consequences of multiple medical problems, physical frailty, and dementia. Access is limited by the ability to pay. Dentists typically apply treatments developed for younger, healthier individuals, with predictably poor results. An article on the dental woes of an aging population [50] best summarizes this dilemma. This report relates an anecdote in which a public health dentist recalls a lecture given 20 years earlier on geriatric treatment during which the speaker presented slides of a patient with substantial evidence of previous treatment with excellent crowns and fillings. However, with gum recession, inflammation, and caries they had led to failure of the fillings and crowns. The patient was a retired dentist who was knowledgeable and had taken care of his teeth throughout his life, but medical problems, side effects of medications, and frailty took their toll. Conventional treatment with a drill was not an option in this case nor is it for many other older adults. The article concludes that with a growing geriatric population, a new standard of dental care was urgent. Nonsurgical treatments such as SDF and ART and FV are effective caries management strategies for older adults that address many of the limitations of traditional treatments.

Our study is a comparative effectiveness study of 2 nonsurgical treatments for addressing both root and coronal caries outcomes in older adults. Previous studies outside of the United States have mainly focused on the prevention and arrest of root caries lesions in community-dwelling older adults [19,21-23]. Both SDF and ART are two relatively inexpensive, evidence-based nonsurgical treatments that can be provided by dental hygienists (dental paraprofessionals) in most states outside of traditional dental clinics to address the unique oral health needs of community-dwelling older adults.

This study adds further to existing knowledge by addressing patient-reported outcomes such as pain and hypersensitivity that are of importance to older adults, as they affect their overall oral health quality of life. In our prior study of older adults living independently in 16 subsidized housing facilities in Northeast Ohio, 62% of the participants reported that their perceived tooth condition was fair/poor [6] with 40% reporting tooth pain and 54% tooth sensitivity. Both treatment strategies have the ability to kill plaque bacteria and strengthen the tooth surface [24-27] and thus address pain and sensitivity. SDF, in particular, has been cleared by the FDA as a tooth desensitizer.

This comparative trial can also provide needed evidence regarding the effectiveness of these interventions in community-based settings (ie, for public health purposes). Currently, there are no community-based public health interventions for older adults like those for children. For any dental caries preventive/treatment intervention to be useful on a population level, 5 presumed attributes are necessary: pain and infection control, simplicity of use, cost affordability, minimal personnel time and training, and noninvasiveness [51]. Potentially, these nonsurgical interventions have these attributes and can address the limitations of the current clinic-based dental care delivery system which is expensive and ill adapted [52].

### Generalizability

The state requirements for supervision vary considerably. The results of this trial may be applicable to settings in which dental hygienists, or perhaps dental therapists, are permitted to provide services under general supervision outside of traditional clinics and practices. The results are limited by the necessity to select particular dental materials based on our current assessment of their practicality and ease of application. Other materials may behave differently as industry is constantly innovating and changing the materials. Nevertheless, evidence for this class of treatment alternatives can result in more robust treatment guidelines and standards that are broadly generalizable.

## Appendix

Multimedia Appendix 1 Study flyer.

## Abbreviations

ART: atraumatic restorative treatment
FV: fluoride varnish
GEE: generalized estimating equation
GIC: glass ionomer cement
ICDAS: International Caries Detection and Assessment System
SDF: silver diamine fluoride
