# Supplementary figures and images for: Comparative Effectiveness of Two Nonsurgical Treatments to Reduce Oral Health Disparities From Untreated Tooth Decay in Older Adults: Protocol for a Cluster Randomized Trial
Source: JMIR Res Protoc. 2020 Sep 8;9(9):e17840. doi: 10.2196/17840 (PMC7509639; doi:10.2196/17840)

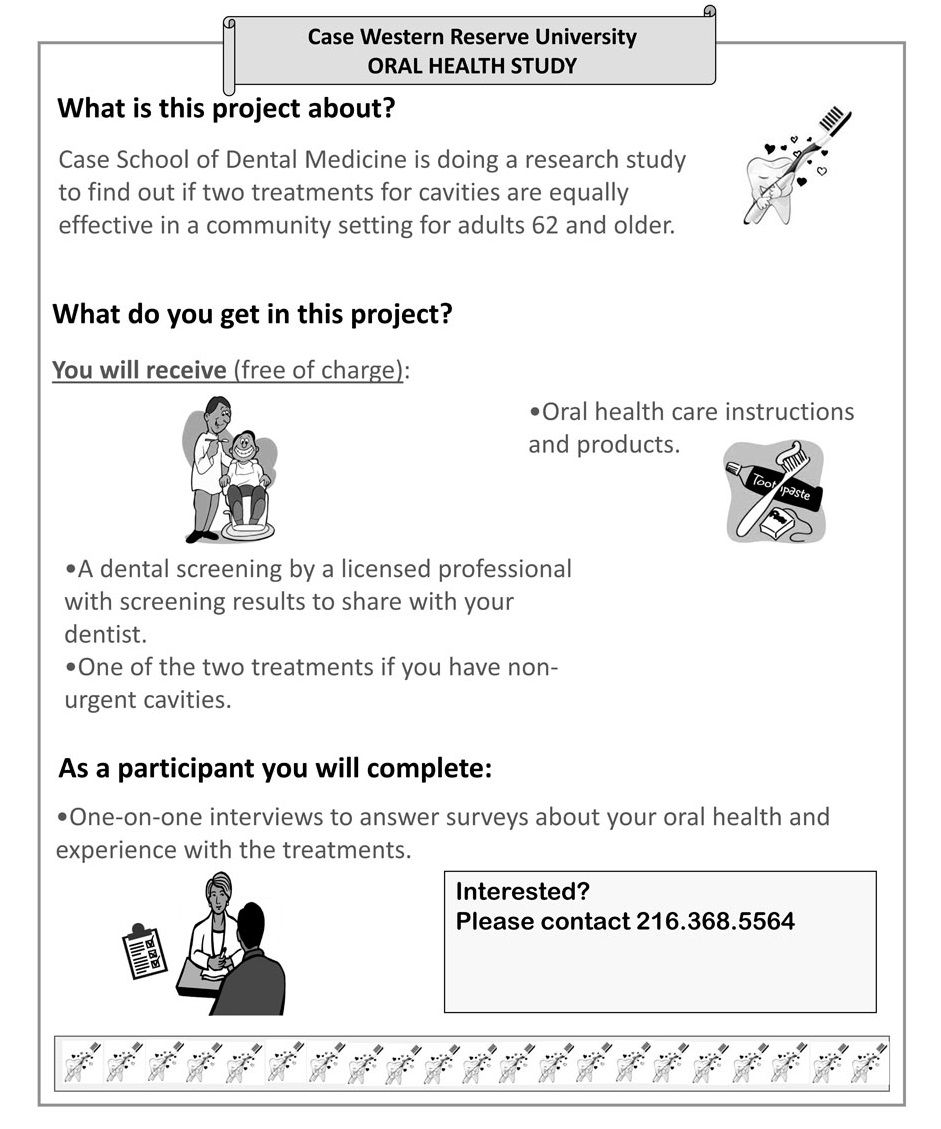

Supplement: Multimedia Appendix 1 [file resprot_v9i9e17840_app1.png]
